# A Trichome-Specific, Plastid-Localized *Tanacetum cinerariifolium* Nudix Protein Hydrolyzes the Natural Pyrethrin Pesticide Biosynthetic Intermediate *trans*-Chrysanthemyl Diphosphate

**DOI:** 10.3389/fpls.2020.00482

**Published:** 2020-04-24

**Authors:** Wei Li, Daniel B. Lybrand, Haiyang Xu, Fei Zhou, Robert L. Last, Eran Pichersky

**Affiliations:** ^1^Department of Molecular, Cellular, and Developmental Biology, University of Michigan, Ann Arbor, MI, United States; ^2^Guangdong Laboratory for Lingnan Modern Agriculture, Genome Analysis Laboratory of the Ministry of Agriculture, Agricultural Genomics Institute at Shenzhen, Chinese Academy of Agricultural Sciences, Shenzhen, China; ^3^Department of Biochemistry and Molecular Biology, Michigan State University, East Lansing, MI, United States; ^4^Center of Plant Functional Genomics, Institute of Advanced, Interdisciplinary Studies, Chongqing University, Chongqing, China; ^5^Department of Plant Biology, Michigan State University, East Lansing, MI, United States

**Keywords:** pyrethrins, Nudix, plastid, trichome, *Tanacetum cineriifolium*

## Abstract

*Tanacetum cinerariifolium* flowers synthesize six pyrethrins that function as effective insecticides. *trans*-Chrysanthemol is an early intermediate in the synthesis of the monoterpene moiety of pyrethrins. Previously, the pyrethrum enzyme chrysanthemyl diphosphate synthase (TcCDS) was shown to catalyze the formation of the prenyl diphosphate compound chrysanthemyl diphosphate (CPP) by condensing two molecules of dimethylallyl diphosphate (DMAPP). Later work also showed that with a low concentration of DMAPP, TcCDS can also remove the diphosphate group to give chrysanthemol. The removal of the phosphate groups from other prenyl diphosphates, such as DMAPP, isopentenyl diphosphate (IPP) and geranyl diphosphate (GPP), was previously shown to occur in two steps. In those cases, the first phosphate group is removed by a member of the Nudix hydrolase protein family, and the second by other unidentified phosphatases. These previously characterized Nudix proteins involved in the hydrolysis of prenyl diphosphates were shown to be cytosolic. Here we report that a plastidic Nudix protein from pyrethrum, designated TcNudix1, has high specificity for CPP and can hydrolyze it to chrysanthemol monophosphate (CMP). *TcNudix1* is expressed specifically in the trichomes of the ovaries, where chrysanthemol is produced. *TcNudix1* expression patterns and pathway reconstitution experiments presented here implicate the TcNudix1 protein in the biosynthesis of chrysanthemol.

## Introduction

**FIGURE 1:**
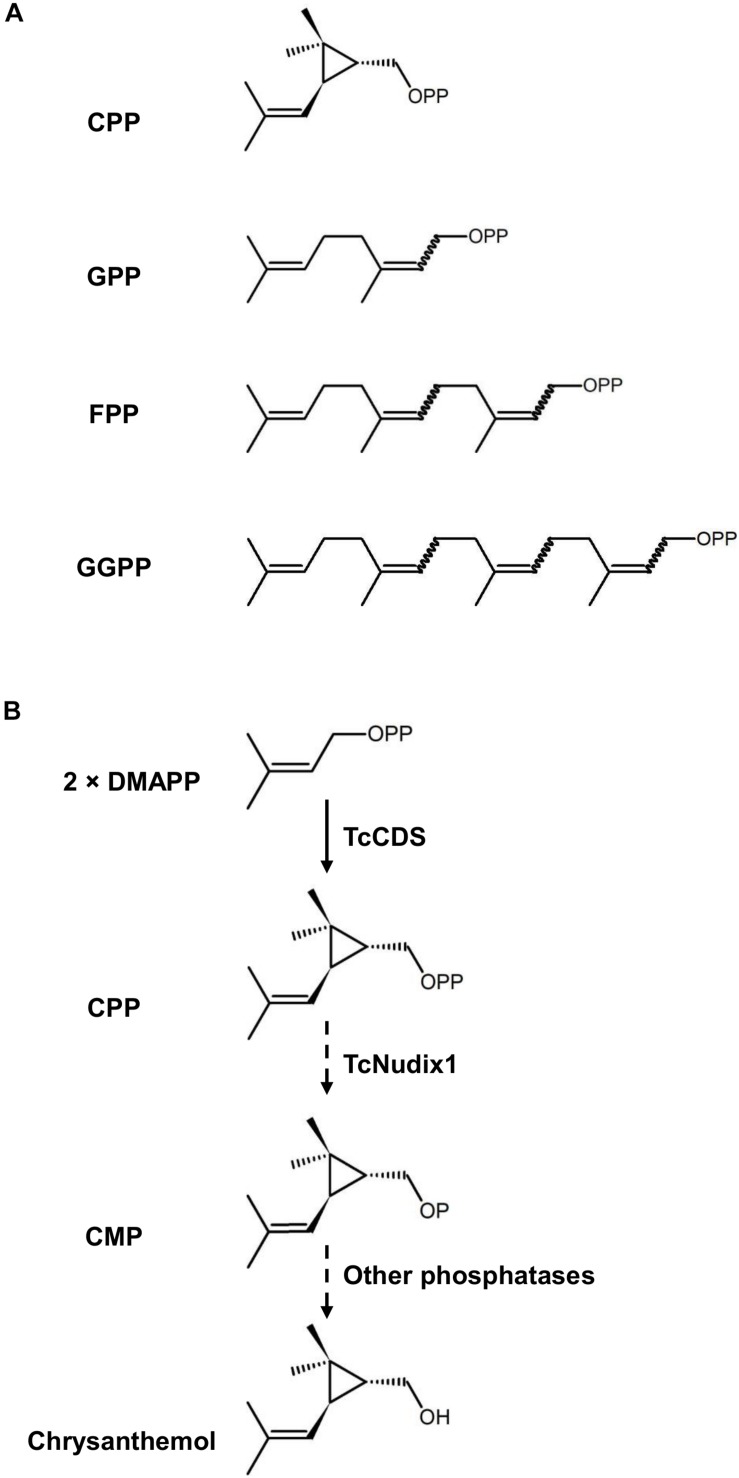
The structures of prenyl diphosphates examined in this study and the hypothetical activity of TcNudix1. **(A)** Chemical structures of chrysanthemyl diphosphate (CPP), geranyl diphosphate (GPP), farnesyl diphosphate (FPP), and geranyl geranyl diphosphate (GGPP). **(B)** TcCDS condenses two molecules of DMAPP to CPP, and TcNudix1 hydrolyzes CPP to chrysanthemyl monophosphate (CMP), which undergoes further hydrolysis to chrysanthemol, the precursor of acid moieties of pyrethrins.

Prenyl diphosphates are intermediates in the synthesis of terpenoid compounds (Pichersky and Raguso, 2018; Karunanithi and Zerbe, 2019). The prenyl moiety of each prenyl diphosphate consists of either a single five-carbon isoprene unit or a linear polymer of this isoprene building block (Figure 1). These prenyl diphosphates serve as terpene synthase substrates, which remove

the diphosphate group and sculpt the prenyl group into the terpene backbone, which is often further modified by additional enzymes (Zhao et al., 2014; Christianson, 2017; Pichersky and Raguso, 2018). However, an alternative route to the removal of the diphosphate group from prenyl diphosphates in the synthesis of terpenes was recently reported (Magnard et al., 2015; Henry et al., 2018). In these cases, the first phosphate group is removed by a cytosolic monophosphatase, which is a member of the Nudix (**nu**cleoside **di**phosphate linked to other moieties **X**) hydrolase family (Mildvan et al., 2005), followed by the hydrolysis of the second phosphate in a reaction catalyzed by another phosphatase (Magnard et al., 2015). Plant Nudix enzymes were further implicated in the regulation of prenyl diphosphate fluxes. For example, the cytosolic AtNudix1 from Arabidopsis catalyzes the hydrolysis of cytosolic dimethylallyl diphosphate (DMAPP) and isopentenyl diphosphate (IPP) to DMAP and IP, respectively, which could be reversed by isopentenyl phosphate kinase (IPK) to regulate the metabolic flux of terpenoid metabolism (Henry et al., 2018).

Chrysanthemyl diphosphate (CPP) is a monoterpene diphosphate, which is an intermediate in the synthesis of the natural pesticides pyrethrins in the flowers of the pyrethrum (*Tanacetum cinerariifolium*) plant (Rivera et al., 2001). CPP is synthesized by the plastid-localized enzyme chrysanthemyl diphosphate synthase (CDS) from the head-to-middle condensation of two DMAPP molecules: this is why CPP is defined as an “irregular” prenyl diphosphate. *In vitro* enzymatic characterization of TcCDS indicated that when the concentration of DMAPP is <300 μM, this enzyme not only catalyzes the formation of CPP, but continues to remove the diphosphate group to give chrysanthemol (Yang et al., 2014). However, a direct proof that TcCDS is completely responsible for the formation of chrysanthemol from CPP *in vitro* is lacking. Here we report that a plastidic Nudix protein in pyrethrum, designated TcNudix1, has high specificity to CPP and is capable of hydrolyzing it to chrysanthemol monophosphate (CMP). The expression of the *TcNudix1* gene is highly specific for the ovarian trichomes where chrysanthemol is known to be produced. TcNudix1 expression patterns and pathway reconstitution experiments presented here provide evidence for the involvement of the TcNudix1 protein in chrysanthemol biosynthesis.

## Results

### Identification of TcNudix1 and Subcellular Localization of the Protein

We previously constructed multiple RNA-seq databases from pyrethrum floral tissues at various developmental stages (Birkett et al., 2000; Xu et al., 2018a). Co-expression analysis using *TcCDS*, which had previously been established to be involved in pyrethrin biosynthesis (Li et al., 2018; Li W. X. et al., 2019), identified a member of the Nudix family within the top 20 contigs whose expression was most highly correlated with TcCDS (contig #12 with a correlation coefficient = 0.9878; Supplementary Table S1). We named this gene *TcNudix1*. While there were at least 56 Nudix hydrolase genes found in our RNA-seq data (albeit not all with full-length gene sequences), none of the other showed a correlation coeeficient of >0.95 to reference gene *TcCDS*.

**FIGURE 2:**
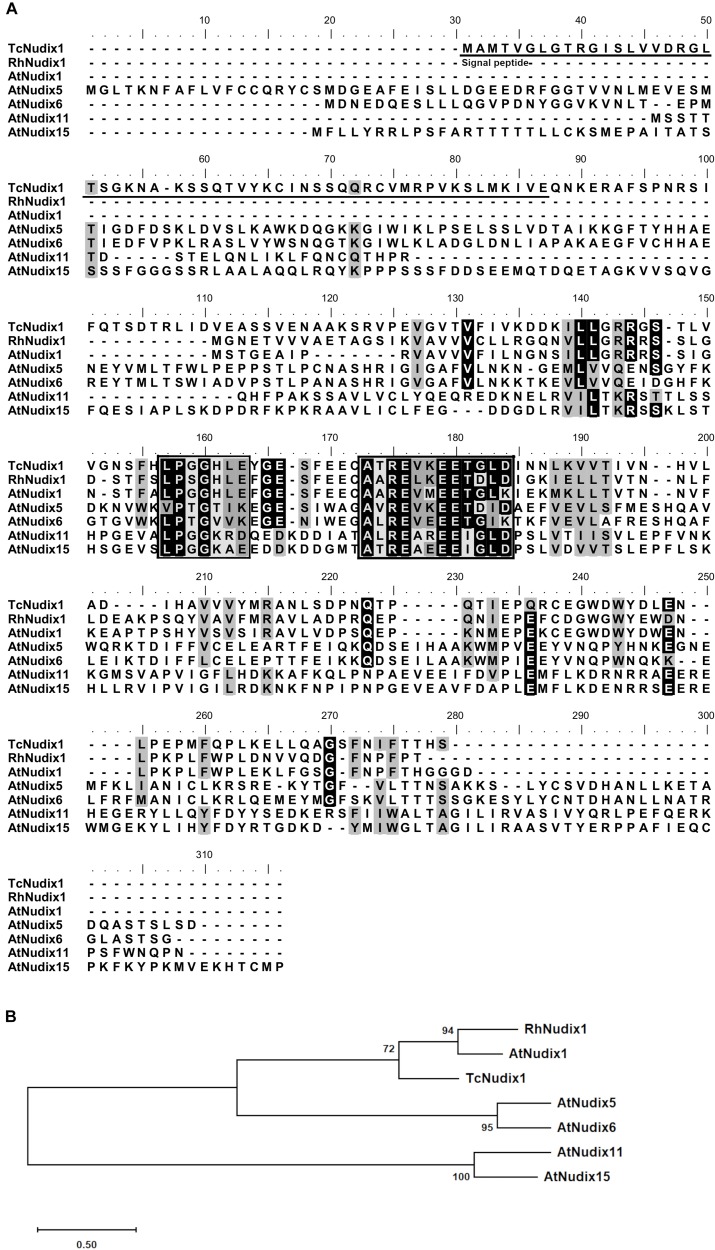
Sequence comparisons of TcNudix1, RhNudix1, AtNudix1, AtNudix2, AtNudix5, AtNudix6, AtNudix11, and AtNudix15. **(A)** Sequence alignment of TcNudix1 and related sequences. Alignment was conducted by ClustalW alignment BioEdit. Black background indicates amino acid identity between sequences and gray background represents amino acid similarity (Threshhold of shading = 60%). The predicted plastid signal peptide of TcNudix1 is underlined. Two boxes show the consensus motifs. **(B)** Maximum-likelihood phylogenetic tree of sequences. RhNudix1 and AtNudix1 were reported to hydrolyze prenyl diphosphate (GPP for RhNudix1 and DMAPP and IPP for AtNudix1). AtNudix11 showed no activity with several prenyl diphosphates but its substrate has not yet been identified (Schilmiller et al., 2012; Henry et al., 2018). No biochemical characterizations of AtNudix2 and AtNudix15 have been performed (Schilmiller et al., 2012; Li J. et al., 2019; Zhou and Pichersky, 2020).

TcNudix1 is a small protein of 229 amino acids with a calculated molecular mass of 25.5 kD. Sequence comparisons of TcNudix1 with RhNudix1 from Rose (*Rosa* × *hybrida*), which hydrolyzes the first phosphate of geranyl diphosphate (GPP), and AtNudix1 from Arabidopsis, which hydrolyzes the first phosphate of IPP and DMAPP, as well as AtNudix5, 6, 11, and 15, for which no prenyl diphosphatase activity was demonstrated (Schilmiller et al., 2012; Henry et al., 2018; Li J. et al., 2019; Zhou and Pichersky, 2020), indicate that all of these sequences share two short conserved sequences in the center of the amino acid chains (boxed areas in Figure 2). However, TcNudix1 shares a much more extensive sequence identity with RhNudix1 and AtNudix1 – the two proteins shown to hydrolyze prenyl diphosphates (63.5% and 50% identities, respectively) – than with the four other Nudix proteins, which have either been shown not to have prenyl diphosphate hydrolyzing activity or have not been biochemically characterized (<20%).

**FIGURE 3:**
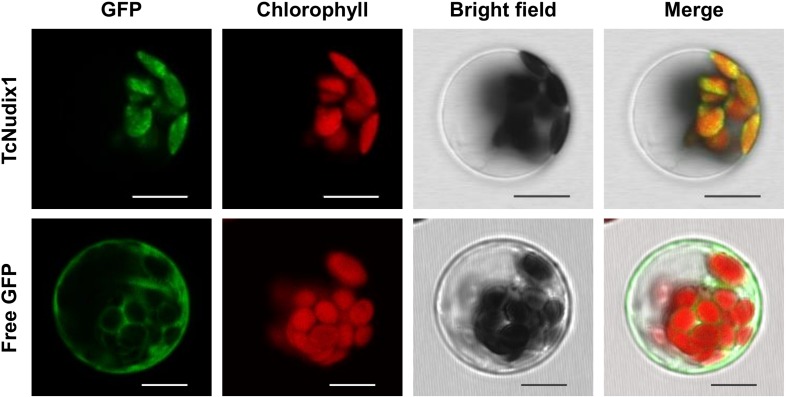
Representative results showing subcellular localization of TcNudix1. Images from left to right are: green fluorescence from GFP, autofluorescence from chloroplasts, bright field under visible light, and overlay of those three images. Images for the same gene are of the same cell. Unfused GFP construct was used as control. Scale bars = 10 μm.

TcNudix1, as well as AtNudix 5, 6, 11, and 15, and a number of other Nudix proteins in sequence databases, have *N*-terminal sequence extensions not found in RhNudix1 and AtNudix1 (Figure 2A). Analysis of TcNudix1 sequence using the ChloroP prediction program suggests that TcNudix1 has a 56 amino acid plastid transit peptide at the *N*-terminus^1^ that targets it to the plastids. To experimentally determine the subcellular localization of TcNudix1, we created a fused gene construct starting with the TcNudix1 ORF and terminating with the GFP ORF, placed it in an expression vector driven by the 35S promoter, and transformed Arabidopsis protoplasts. Confocal microscope images showed that in protoplasts transformed with the TcNudix1-GFP fusion construct, the observed GFP signal overlapped with the chloroplast autofluorescence signal, but in protoplasts transformed with a stand-alone GFP ORF the GFP signal was present in the cytosol (Figure 3).

### Tissue-Specific Expression of *TcNudix1*

**FIGURE 4:**
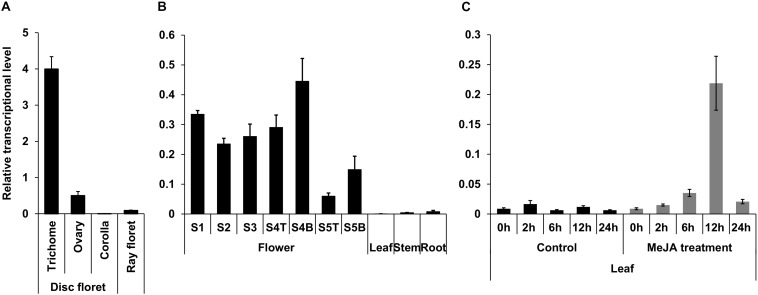
Tissue specific expression of TcNudix1. **(A)** RT-qPCR analysis of *TcNudix1* transcript levels in different parts of stage-3 flowers. **(B)** RT-qPCR analysis of *TcNudix1* transcript levels in different developmental stages of flowers, leaf, stem and root (“T”: ray floret, “B”: disk florets). **(C)** RT-qPCR analysis of 2-week-old leaves treated with MeJA. (Data are presented as means ± SD, *n* = 3 or 4).

Genes participating in the early steps of the synthesis of the terpene moiety of pyrethrins are highly expressed in the trichomes of ovary, show increased expression in later stages of flower development, and are induced by MeJA in leaves (Li et al., 2018). Analysis of TcNudix1 gene expression (Figure 4) shows that *TcNudix1* is also highly specific to trichomes (Figure 4A), and its developmental stage expression pattern is similar to other genes involved in the early steps of the synthesis of the terpene moiety of pyrethrins (Figure 4B). Furthermore, *TcNudix1* expression is also induced by MeJA (Figure 4C).

### Characterization the Hydrolysis Activity of TcNudix1

**FIGURE 5:**
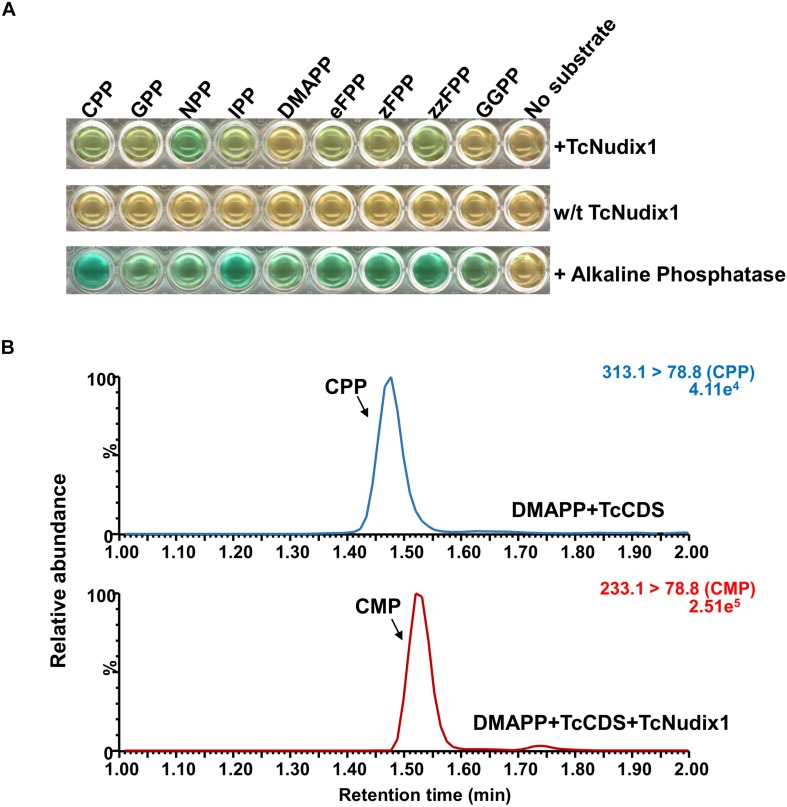
Activity of TcNudix1 with prenyl diphosphate substrates. **(A)** A colorimetric assay in which green color indicate positive results. In each well, 5 μg of purified TcNudix1was incubated with 10 μM of the indicated substrate for 30 min. **(B)** LC-MS analysis of conversion of CPP to CMP catalyzed by TcNudix1. DMAPP was incubated with TcCDS to generate CPP as the substrate for TcNudix1, then TcNudix1 was added to produce CMP, which was detected with LC-MS under MRM mode; precursor ion and product ion for CPP are 313.1 and 78.8, respectively, (313.1 > 78.8), and the ions for CMP are 233.1 and 78.8.

To characterize TcNudix1 enzymatic activity, we produced a recombinant protein (lacking the part of the ORF encoding the transit peptide and containing a His-tag at the *C*-terminus) in *Escherichia coli*, purified it by affinity chromatography (Supplementary Figure S1), and tested its activity with various prenyl diphosphate substrates (Figure 5A). TcNudix1 showed highest levels of activity with the *cis*-prenyl diphosphates NPP and zFPP as well as with CPP. It was less active with IPP, GPP, and eFPP, and showed no activity with DMAPP and GGPP (Figure 5A and Supplementary Table S2). TcNudix1 hydrolyzed CPP to CMP (Figure 5B) with a *K*_*m*_ value of 0.137 ± 0.05 μM.

**FIGURE 6:**
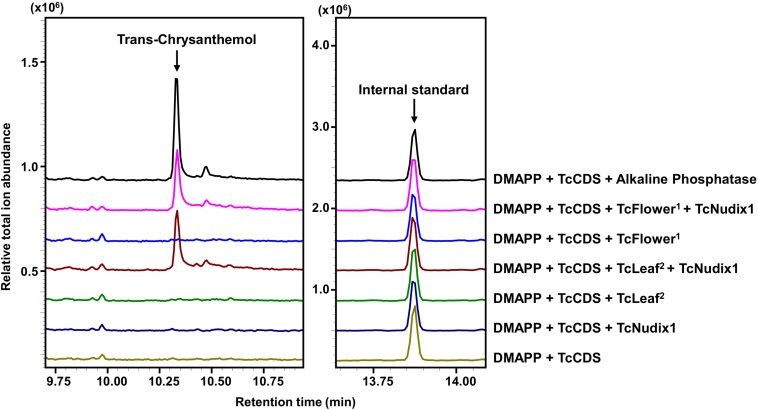
Complete conversion of CPP to chrysanthemol by TcNudix1 and additional phosphatase(s) from pyrethrum tissues. DMAPP was incubated with TcCDS to generate CPP, followed by the addition of TcNudix1, TcNudix with crude flower protein (TcFlower^1^), only crude flower protein extract, TcNudix1 with crude leaf protein (TcLeaf^2^), and only crude leaf protein. Chrysanthemol was detected by GC-MS. DMAPP and TcCDS with alkaline phosphatase treatment was used as positive control. Tetradecane was added as internal standard.

We further tested for the presence of a second phosphatase in pyrethrum flowers and leaves by performing *in vitro* coupled assays that included DMAPP and TcCDS (to generate CPP) and TcNudix1 alone or both TcNudix1 and crude protein extracts from pyrethrum flowers or leaves (Figure 6). After overnight incubation, the reaction products were extracted and analyzed by GC-MS (Figure 6). Chrysanthemol was detected when TcCDS, TcNudix1 and crude protein from pyrethrum flowers or leaves were included. No chrysanthemol was detected when CPP was incubated with TcNudix1 alone without leaf or floral crude protein extracts, or with floral or leaf crude protein extracts without added TcNudix1. The most likely explanation for the latter observation is that concentration of TcNudix1 in the crude extract, while high relative to other proteins (assumed from the transcript data), is still much lower than the concentration of the added purified recombinant TcNudix1 protein, so that the two-step reaction of converting the exogenously added CPP to chrysanthemol is too slow to yield a detectable product.

### Heterologous Co-Expression of *TcNudix1* and *TcCDS* in Tomato

**FIGURE 7:**
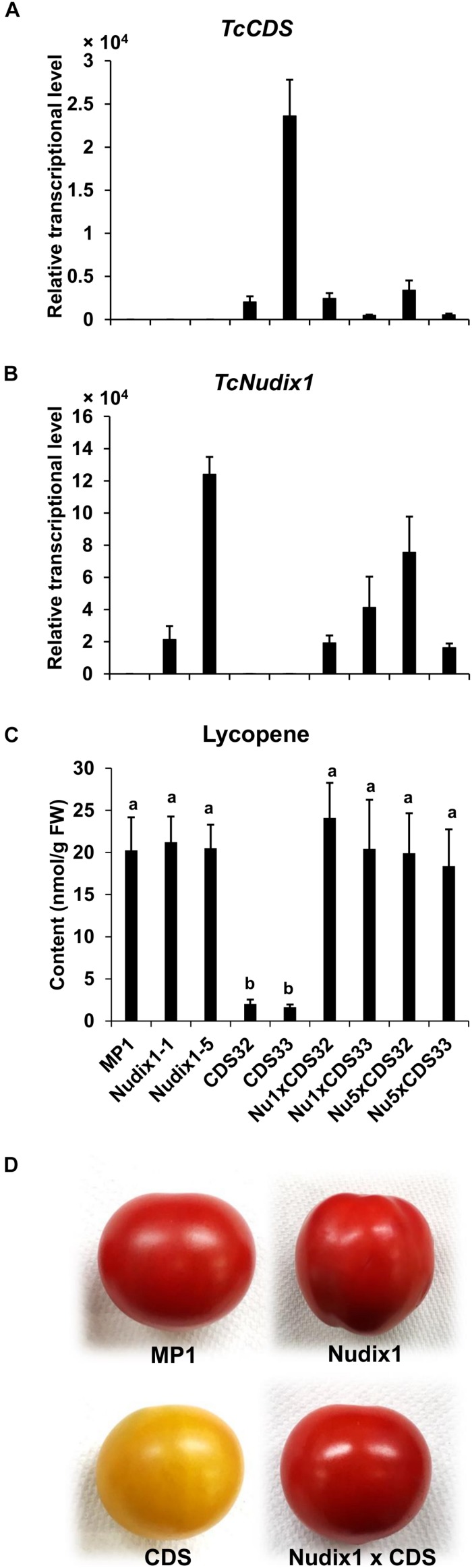
Coexpressing *TcNudix1* with *TcCDS* in tomato fruit restores wild-type lycopene levels. **(A)** RT-qPCR analysis of TcNudix1 transcript levels in fruits of tomato transgenic lines express TcCDS (CDS32/33), TcNudix1 (Nudix1-1/5) and coexpression lines (Nu1/5 × CDS32/33). **(B)** RT-qPCR analysis of TcNudix1 transcript levels in fruits of each lines. **(C)** Lycopene content in fruits of each lines. **(D)** Color of mature fruits from different lines.

We previously expressed *TcCDS* in tomato fruit under the control of the polygalacturonase (PG) promoter. We observed that the amount of the red pigment lycopene produced in the tomato fruit expressing TcCDS was greatly reduced (Xu et al., 2018a), likely because CPP made in these fruits competitively inhibits the activity of geranylgeranyl diphosphate synthase (GGPPS), an enzyme that catalyzes the formation of GGPP, an intermediate in lycopene biosynthesis, from DMAPP, and IPP (Gutensohn et al., 2013; Xu et al., 2018a). To test if TcNudix1 can hydrolyze CPP in tomato fruit expressing *TcCDS* and thereby relieve the inhibition of GGPPS, we obtained transgenic tomato plants expressing TcNudix1 under the control of the PG promoter and crossed these lines to the *TcCDS*-overexpressing lines and measured the concentration of lycopene (Figure 7). Plants expressing *TcCDS* alone (Figures 7A,B) indeed showed greatly decreased levels of lycopene biosynthesis (Figures 7C,D). In contrast, plants expressing *TcNudix1* in addition to *TcCDS* (Figures 7A,B) had wild-type levels of lycopene (Figures 7C,D).

## Discussion

The two previously identified Nudix proteins involved in terpenoid metabolism – a GPP monohydrolase in rose and an IPP mono-phosphate hydrolase in Arabidopsis – were shown to be cytosolically localized. Here we present evidence that TcNudix1 is a plastid-localized mono-phosphate hydrolase that favors the *cis*-prenyl diphosphates NPP and zFPP and the irregular prenyl diphosphate CPP. It displays lower activities with some *trans*-prenyl diphosphates and negligible activity with DMAPP and GGPP.

Chrysanthemyl diphosphate is an intermediate in the synthesis of chrysanthemic and pyrethric acids, which are combined with jasmone-derived alcohols in the biosynthesis of the natural pyrethrin insecticides (Rivera et al., 2001). All of the steps in the biosynthesis of these acids, with the exception of the ultimate methylation step in the synthesis of pyrethric acid, occur in the trichomes of the developing achenes in pyrethrum flowers (Ramirez et al., 2012). These steps include the condensation of two DMAPP molecules to synthesize CPP by the plastid-localized and trichome-specific TcCDS. It was also shown that TcCDS, under low DMAPP concentrations, could also catalyze a second reaction – the formation of chrysanthemol from CPP. The *TcNudxi1* gene is highly and specifically expressed in the trichomes of the developing achenes (Figure 4), and its high affinity for CPP suggests that it contributes to its conversion to CMP. Results of heterologous expression of TcNudix1 in tomato fruit also indirectly indicate that TcNudix1 is capable of hydrolyzing CPP, because the CPP inhibition of GGPPS was eliminated, with the result that lycopene biosynthesis levels increased (Figure 7). Since CPP is synthesized from two DMAPP molecules (Figure 1), it is noteworthy that DMAPP is not a substrate of TcNudix1.

Since TcNudix1 removes only the terminal diphosphate from CPP, it must work together with other phosphatase(s) to finish the ultimate production of chrysanthemol. Assays with extracts from both flowers and leaves of pyrethrum demonstrate that a hydrolase capable of doing so is present in these tissues (Figure 6). It is likely, but not yet proven, that the phosphatase that removes the phosphate from CMP is also plastid-localized, but this needs to be examined. Because the next step in the pyrethrin pathway – the conversion of chrysanthemol to chrysanthemal – was shown to occur in the cytosol (Xu et al., 2018b), it remains to be shown how chrysanthemol (or CMP) is transported to the cytosol. Overall, the evidence presented here suggests that TcNudix1 may contribute to the formation of chrysanthemol in the synthesis of pyrethrins, and this result extend the involvement of plant Nudix proteins in the regulation of prenyl diphosphate flux to the plastidic compartment.

## Materials and Methods

### Plant Growth Condition

Pyrethrum (*T. cinerariifolium*) plants were grown in growth chambers on soil at 25°C during the 16 h light period and at 20°C during the 8 h dark period. Tomato (*Solanum lycopersicum*) plants were grown in growth chambers on soil at constant 22°C at a 16 h light/8 h dark regime.

### Co-expression Analysis

Co-expression analysis was performed by comparing transcriptional data of all genes with *TcCDS* via Pearson correlation using SPSS statistics^2^. The program ranks the similarity levels of expression patterns.

### Subcellular Localization

The open reading frame (PRF) of *TcNudix1* was integrated to plasmid pEZS-NL to create an PRF fused to GFP. The construct was mobilized into Arabidopsis protoplast cells for confocal microscopy examination.

### *In vitro* Expression and Enzymatic Assay

The ORF of *TcNudix1* without the *N*-terminal 56 amino acid transit peptide and stop codon was integrated into the vector pET28a, then the construct was mobilized to *E coli.* BL21(pLySs). The bacteria were cultured in 500 ml LB to OD600 = 0.6 at 37°C, and protein expression was induced by 0.5 mM IPTG and 16°C shaking. After overnight incubation, the induced bacterial culture was centrifuged, and bacteria were resuspended in wash buffer (50 mM Tris, pH8.0, 300 mM NaCl, 20 mM imidazole, and 10 mM 2-ME). Bacteria cells were broken up by ultrasonication (3 s sonicate with 6 s break, 200 times). Qiagen Ni-NTA agarose (0.5 ml) was added to each lysate and incubated for 1 h to let protein bind, then the mixture was poured into Qiagen 1 ml polypropylene columns and the agarose was washed with 50 ml wash buffer. Finally the purified protein was eluted with elution buffer (50 mM Tris, pH8.0, 300 mM NaCl, 250 mM imidazole, and 10 mM 2-mercaptoethanol).

The purified TcNudix protein was incubated with CPP or other prenyl diphosphate [CPP was made following the reference of Rivera et al. (2001)]. The 50 μl reactions contained: 100 mM Tris (pH 7.5), 5 mM MgCl_2_, and 1 μM TcNudix1 protein and various concentration of prenyl diphosphate (0.2–10 μM). The reaction solution was extracted directly for LC-MS or incubated overnight with crude protein extracts from flowers or leaves (not induced by MeJA) for GC-MS (see below). When measuring kinetic parameters, purified TcNudix1 was incubated with CPP from 0.1 to 5 μM for 5 min reaction and the decrease in the CPP peak as measured by LC-MS was calculated.

The colorimetric assay for monophosphatase activity of TcNudix1 with different prenyl diphosphate substrates was performed by mixing 7 micrograms of purified TcNudix1 with 10 μM each substrate in 50 μl reaction containing 100 mM Tris (pH 7.5) and 5 mM MgCl_2_. Each reaction was incubated for 30 min at room temperature, then 100 μl BioMol Green Reagent (Enzo Life Science) was added and incubated for 30 min for the color to develop. The yellow malachite green molybdate in this reagent binds to free orthophosphate and forms a green complex that absorbs at 620–640 nm.

### LC-MS Analysis

Enzymatic reactions of TcNudix1 were terminated with the addition of 50 μl chloroform and 50 μl water followed by vortexing. After centrifugation at maximum speed for 5 min, the aqueous phase was used for LC-MS analysis.

Samples were run on Waters Micromass Quattro Premier Mass Spectrometer LC-MS/MS System with Supelcosil LC-18 column. The program was set as hold 99% mobile phase A (0.05% triethylamine) and 1% mobile phase B (50% acetonitrile, 50% isopropanol, and 0.05% triethyamine) for 0.5 min, then convert to 1% A and 99% B in a linear gradient from 0.5 to 3 min, and hold 1% A for 1 min. Peaks were detected in MRM mode (multiple reaction monitoring). The precursor ion for CPP and GPP is 313.1, for CMP and GMP is 233.1, for FPP is 381.1, for FMP is 301.1, for GGPP is 449.2, for GGMP is 369.2, product ion for all compounds is 78.8.

### GC-MS Analysis

Plant tissue (pyrethrum stage 3 flowers) were ground in liquid nitrogen and incubated on ice with extraction buffer containing 100 mM Tris (pH7.5), 1% (w/v) Polyvinylpolypyrrolidone and 10 mM 2-mercaptoethanol to obtain crude protein extract. TcNudix1 reactions were incubated with 10 μl plant crude protein overnight at room temperature and extracted with 50 μl MTBE for GC-MS analysis. Samples were run on Shimadzu GC-MS-QP5000 with TR-5MS column. The program was conducted as following; 50°C for 5 min, 50°C to 280°C for 10°C/min, 28°C hold for 5 min.

### Lycopene Measurements

Measurements of lycopene were performed as previously described (Gutensohn et al., 2014). Fruit tissue was homogenized and extracted with acetone/hexane (4/6), absorbance at 663, 645, 505, and 453 were measured by spectrophotometer. Content of lycopene = −0.0458 × A_663_ + 0.204 × A_645_ + 0.372 × A_505_ − 0.0806 × A_453_ (mg/100 ml).

### RT-qPCR

RNA extraction, reverse transcription reaction and q-PCR assays were conducted as previously described (Li W. X. et al., 2019).

### Tomato Transformation

The ORF of *TcNudix1* was amplified and integrated into plasmid pBin19 with the fruit-specific PG promoter and tomato MP1 plants were transformed as previously described (Xu et al., 2018a). Transgenic tomato TcNudix1 lines were crossed with transgenic lines containing a *TcCDS* gene under the control of the PG promoter to obtain *TcCDS* × *TcNudix1* lines.

## Primers

All primers used in this study were listed in Supplementary Table S3.

## Data Availability Statement

The sequence of TcNudix1 has been submitted to NCBI GenBank under accession number of MT126704.

## Author Contributions

WL, HX, RL, and EP designed the experiments; WL, DL, and FZ conducted the experiments; WL, RL, and EP wrote the article; all authors edited the manuscript.

## Conflict of Interest

The authors declare that the research was conducted in the absence of any commercial or financial relationships that could be construed as a potential conflict of interest.

## Footnotes

1 http://www.cbs.dtu.dk/services/ChloroP/

2 https://www.ibm.com/products/spss-statistics
