# Supplementary material for: A Trichome-Specific, Plastid-Localized Tanacetum cinerariifolium Nudix Protein Hydrolyzes the Natural Pyrethrin Pesticide Biosynthetic Intermediate trans-Chrysanthemyl Diphosphate
Source: Front Plant Sci. 2020 Apr 24;11:482. doi: 10.3389/fpls.2020.00482 (PMC7194074; doi:10.3389/fpls.2020.00482)
Supplement: Supplementary file 1 [file Data_Sheet_1.PDF]

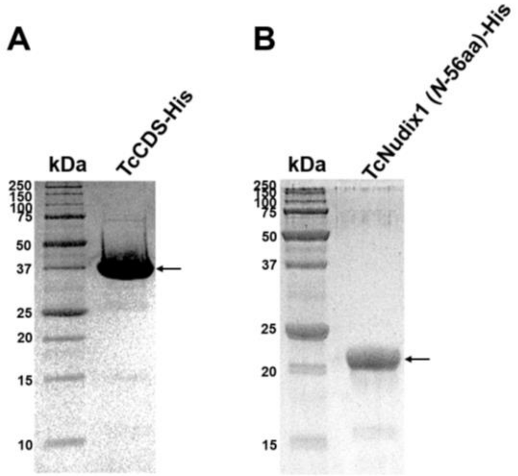

Supplemental figure S1. SDS-PAGE image of purified TcCDS **(A)** and truncated TcNudix1**(B)**.

Supplemental table S1. Correlation analysis

Rank	Gene	Pearson Correlation	P value (two-tailed)	Predicted function
	CDS	1		
1	TRINITY_DN91754_c0_g2_i1	0.999098465	1.8306E-09	
2	TRINITY_DN158798_c1_g5_i1	0.996393869	1.1692E-07	
3	TRINITY_DN149780_c0_g1_i2	0.994524669	4.08683E-07	Glutathione S-transferase
4	TRINITY_DN159661_c0_g4_i9	0.994044212	5.25794E-07	Heat_shock_protein
5	TRINITY_DN147751_c0_g1_i2	0.993810671	5.90001E-07	
6	TRINITY_DN129539_c0_g1_i1	0.993336904	7.3586E-07	Lipase
7	TRINITY_DN128614_c0_g1_i1	0.992671839	9.78442E-07	Acyl-transferase
8	TRINITY_DN75102_c0_g2_i1	0.991266721	1.65433E-06	
9	TRINITY_DN135414_c0_g1_i1	0.9895933	2.79565E-06	Laccase-22_Multicopper_oxidase
10	TRINITY_DN182299_c0_g1_i1	0.989122858	3.19106E-06	
11	TRINITY_DN159654_c0_g1_i3	0.98809509	4.18054E-06	
12	TRINITY_DN158987_c4_g2_i1	0.987774188	4.52671E-06	Nudix1
13	TRINITY_DN4250_c0_g1_i1	0.987757184	4.54556E-06	
14	TRINITY_DN130811_c0_g1_i1	0.987661988	4.65209E-06	Pectinesterase
15	TRINITY_DN142284_c0_g2_i7	0.986848332	5.63104E-06	Protein_phosphatase
16	TRINITY_DN143344_c0_g1_i1	0.986703478	5.81853E-06	Pinoresinol-lariciresinol_reductase
17	TRINITY_DN157112_c1_g1_i1	0.986141549	6.58505E-06	
18	TRINITY_DN157112_c1_g1_i1	0.986141549	6.58505E-06	
19	TRINITY_DN151384_c1_g2_i1	0.985915932	6.91075E-06	aldehyde dehydrogenase
20	TRINITY_DN159641_c5_g2_i3	0.984992529	8.35529E-06	
…	…	…	…	

Supplemental table S2. Substrate specificity of TcNudix1 to CPP, GPP and GGPP.

Substrate[a]	Relative activity
Chrysanthemyl diphosphate (CPP)	100%[b]
Geranyl diphosphate (GPP)	11.26 ± 3.08%
Farnesyl diphosphate (FPP)	10.01 ± 0.78%
Geranyl geranyl diphosphate (GGPP)	Not Detected

a. Concentration of substrate is 0.1 μM
b. 100% represent 2.93 nmol/s/μmol protein

Supplemental table S3. Primers used in this study

Name	Sequence
pET28-TcNudix1 (-56aa)-F	GGCGAATTCCAAAACAAGGAACGAGCATTTTC
pET28-TcNudix1 (-56aa)-R	GGCGTCGACTCAAGAATGAGTAGTGAAAATATTG
Nudix1 RT1-F	CTCGGGGAAGAATGCTAAATCA
pEZS-NL-Nudix1-F	GGCGAATTCATGGCGATGACAGTTGGTTTAGG
pEZS-NL-Nudix1-R	GGCGGATCCCAAGAATGAGTAGTGAAAATATTG
Nudix1 RT1-R	ACACCGTAACCCCAACCTCTG
Nudix1 RT2-F	TCGCGGCTCAACACTCGT
Nudix1 RT2-R	GGCGTCTGATTTGGGTCTGA
pBin19-Nudix1-F	GGCGGATCCATGGCGATGACAGTTGGTTTAGG
pBin19-Nudix1-R	GGCACTAGTTCAAGAATGAGTAGTGAAAATATTG
MCPI-F	CGCGGATCCATCCTGAGCTAGAAGTTATGACCGTTTG
MCPI2nd-overlap (Nudix)-R	AACTGTCATCGCCATATATTATGTGATGCTACTTTGATTGG
Nudix F	ATGGCGATGACAGTTGGTTTAGG
Nudix (NOS)-overlap R	AATGTTTGAACGATCTCAAGAATGAGTAGTGAAAATATTG
NOS-F	GATCGTTCAAACATTTGGCAATAAA
NOS-R	GCCGAATTCGATCTAGTAACATAGATGACACCGCG
